# Integrated transcriptomic and metabolomic analyses reveal the regulatory mechanism of subcutaneous fat deposition in Baicheng Oil chickens

**DOI:** 10.1016/j.psj.2026.106894

**Published:** 2026-04-02

**Authors:** Xiaoyu Zhao, Yang Yao, Haiying Li, Herong Liao, Wei Dong, Yingping Wu

**Affiliations:** a College of Animal Science, Xinjiang Agricultural University, Urumqi, 830052, China; b Xinjiang Noach Baicheng Oil Chicken Development Co., Baicheng, 842300, China

**Keywords:** Subcutaneous fat, Meat quality, Tanscriptome, Metabolism, Baicheng oil chicken

## Abstract

To investigate the differences in meat quality between high **(FH)** and low **(FL)** subcutaneous fat thickness in Baicheng oil chickens and their possible regulatory mechanisms, this study jointly analysed the transcriptome and metabolome characteristics of the liver and subcutaneous fat tissue of Baicheng oil chickens. The results showed that there were significant differences in muscle fatty acid content between the FH and FL groups. Through a combined analysis of the transcriptome and metabolome of the liver and subcutaneous fat tissue of Baicheng oil chickens, differentially expressed genes **(DEGs)** and metabolites such as ArfGAP with RhoGAP domain, ankyrin repeat and PH domain 2 (**ARAP2)**-N-Acetylhistidine, phosphoenolpyruvate carboxykinase 1 (**PCK1)**-ACar 16:1, and PCK1-ACar 20:2 were identified as having significant correlations, synergistically regulating the lipid metabolism process in chickens. This study contributes to an in-depth understanding of the mechanisms of fat regulation and the formation of excellent meat quality traits in local chickens and provides a basis for the identification of biomarkers of breeding value.

## Introduction

Chicken is an important source of animal protein in the daily dietary structure of humans. As the dietary structure of human beings continues to improve, consumers have higher requirements for the quality of chicken meat. However, improving meat flavour has become an urgent challenge for the global poultry industry. The formation of flavor in chicken meat is closely linked to the process of fat deposition, which not only affects the tenderness and juiciness of the meat but also plays a key role in the formation of flavor (Jin et al., 2020). Baicheng oil chicken is a famous local breed for both meat and eggs in China. It is named oil chicken because there is a layer of fat between its skin and muscle. Baicheng oil chicken has delicious meat and unique flavor, which is a good material for high-quality chicken breeding, but it is lack of accurate analysis of its subcutaneous fat formation and meat quality traits.

Metabolomics is the sum of all low molecular weight metabolites produced in a given organism or cell at a given biological stage and is used to describe the interactions of these metabolites within the organism and the organism's response to the external environment (Si, et al., 2023). With the development of high-throughput detection technologies such as liquid chromatography-mass spectrometry, metabolomics has been widely used in biological studies such as fat regulation and meat quality analysis (Dal Bosco et al., 2012; Liu, et al., 2021). Zhao et al. (2025) integrated lipidomic and non-targeted metabolomic analyses and found that differential metabolites such as lysoPS 18:1, lysoPC 20:3, and lysoPC 18:2 play a dominant role in intramuscular fat deposition, which ultimately improves the meat flavor of Jingyuan chickens. RNA sequencing (RNA-Seq) analyses estimate the mRNA expression profiles of genes in an organism at a given point in time, which can help to understand the expression levels of genes under different conditions, thus revealing the impact of gene regulation on metabolite changes (Hirai et al., 2004). The combined use of metabolomics and transcriptomics data contributes to a better understanding of the mechanisms of complex trait formation. In poultry, the regulatory process of intramuscular fat and abdominal fat formation in poultry meat was revealed by combined transcriptomic and metabolomic analyses, which provided valuable data references for improving chicken meat quality (Wang et al., 2023). The above studies have improved the understanding of the mechanisms of fat regulation and meat flavor formation in poultry, but only a few poultry breeds are involved, and the excellent meat characteristics of local poultry breeds need to be accurately characterized, and the mechanisms of their formation still need to be explored.

Therefore, in this study, the transcriptome and metabolome characteristics of the liver and subcutaneous adipose tissue of Baicheng oil chickens with significant differences in subcutaneous fat thickness were analyzed, and the key genes and metabolites related to the regulation of subcutaneous fat in Baicheng oil chickens were screened, so as to provide valuable references for further research on the molecular mechanisms of the formation of excellent meat traits by adipose regulation in Baicheng oil chickens.

## Materials and methods

### Ethics statement

Sample collection involved were approved by the Animal Welfare and Ethics Committee of Xinjiang Agricultural University, Urumqi, Xinjiang, China (Approval number 2023007).

#### Test animals

In this study, the same batch of Baicheng oil chicken hens with the same level of feeding management and similar body weight were selected for slaughtering, and the subcutaneous fat thickness was measured using vernier calipers (the skin on both sides was peeled off from the tail root tangent upwards along the first tangent line, and the thickness of the sebum at this point was measured using vernier calipers). Based on the subcutaneous fat deposition, individuals with highly significant differences in subcutaneous fat thickness were selected to form a high subcutaneous fat deposition group (FH group, subcutaneous fat thickness of 13.01±1.57 mm) and a low subcutaneous fat deposition group (FL group, subcutaneous fat thickness of 9.26±1.64 mm), with 10 chickens in each group.

#### Muscle flavouring substances

The ipsilateral pectoralis and hamstring muscles of the FH and FL groups were collected and stored at −80°C for the determination of intramuscular fat content and muscle flavor. Muscle flavor indicators included amino acid content and fatty acid content, and muscle amino acid content was determined by an amino acid analyzer by the method of GB 5009.124-2016 ‘Determination of Amino Acids in Food Safety National Standard for Foods’; muscle fatty acid content was determined by an amino acid analyzer by the method of GB 5009.168-2016 ‘Determination of Fatty Acids in Food Safety National Standard for Foods’. The fatty acid content of muscle was determined by gas chromatography according to the method of GB 5009.168-2016 ‘Determination of fatty acids in foodstuffs’ of China; the fatty acid content of muscle was determined by gas chromatography. Sichuan Willtest Technology Co., Ltd. (Chengdu, China) was responsible for the completion of the specific process.

#### Morphological analysis of liver and subcutaneous adipose tissue

Liver and subcutaneous adipose tissue were collected from FH and FL groups, and the surfaces were rinsed using saline and dipped dry with skimmed cotton wool, fixed in 4% paraformaldehyde solution, and embedded in paraffin. Sections were serially cut using a slicer with a thickness of 5 μm, and the sections were stained using hematoxylin-eosin **(HE)**. Samples for frozen sections were placed in a 30% sucrose solution for dehydration and OCT embedding. Sections were serially sectioned using a slicer at a thickness of 8 μm and stained using Oil Red O staining solution. After fixing the sections, the liver and subcutaneous adipose tissue sections were observed and photographed under an electron microscope; five fields of view were randomly selected for each sample section, and the subcutaneous adipocyte cross-sectional area (μm^2^) was calculated using ImageJ software (https://imagej.net/software/imagej/).

#### Transcriptome and metabolome sample collection

Liver and subcutaneous adipose tissue were collected from FH and FL groups, and each sample was divided into three tubes and immediately stored at −80°C for transcriptome and metabolome analysis. Liver tissues for transcriptome analysis were labeled FHZG and FLZG, and subcutaneous adipose tissues were labeled FHZP and FLZP, numbered 1-10 sequentially. liver tissues for metabolome analysis were labeled FHG and FLG, and subcutaneous adipose tissues were labeled FHP and FLP, numbered 1-10 sequentially.

#### RNA extraction, sequencing, and transcriptome data analysis

Total RNA extraction and RNA-seq services were provided by Novogene Co., Ltd. (Beijing, China). The Illumina NovaSeq 6000 sequencing platform was used to construct libraries and perform paired-end sequencing. Sequencing data from each sample were aligned to the chicken (Gallus gallus) genome (Ensembl release 108) using HISAT2 (version 2.0.5). Gene expression levels were quantified as FPKM (fragments per kilobase of transcript per million fragments mapped). Differential expression analysis was performed using DESeq2 (version 1.20.0). Raw read counts were first normalized to account for sequencing depth, followed by hypothesis testing to calculate p-value. To control for false positives due to multiple comparisons, the Benjamini-Hochberg false discovery rate (FDR) correction was applied to obtain adjusted p-values (padj). Differentially expressed genes (DEGs) were identified using a threshold of |log_2_(Fold Change)| ≥ 1 and *P*-value < 0.01. Gene Ontology (GO) and KEGG enrichment analyses were performed for the DEGs, and pathways with *P* < 0.01 were considered significantly enriched.

#### Real-time fluorescence quantitative PCR validation

Ten DEGs were randomly selected from the RNA-seq results for real-time fluorescence quantitative PCR validation. Tissue samples were ground in liquid nitrogen and total RNA was extracted according to the protocol of the TRIzol kit (Takara, Dalian, China). β-actin was selected as the internal reference gene, and primers for RT-qPCR were all designed using Oligo 6.0 software (Table S1). The total reaction system was 20 μL, and the cycling procedure was as follows: first pre-denaturation at 95°C for 5 min, followed by 40 cycles of denaturation at 95°C for 10 s and extension at 60°C for 30 s. The melting curve was 60°C→95°C, with the temperature increasing by 0.3°C every 15 s. The relative expression of genes was analyzed by the 2^-ΔΔCT^ method.

#### Untargeted metabolomics profiling

Untargeted metabolomics services were provided by Novogene Co., Ltd. (Beijing, China). Tissues were individually ground with liquid nitrogen and the homogenate was resuspended with prechilled 80% methanol by vigorous vortexing. The samples were incubated on ice for 5 min and then centrifuged at 15,000 g, 4°C for 20 min. An aliquot of the supernatant was diluted with LC-MS grade water to a final methanol concentration of 53%. The samples were subsequently transferred to a fresh Eppendorf tube and centrifuged at 15,000 g, 4°C for 20 min. Finally, the supernatant was injected into the LC-MS system for analysis (Want et al., 2013). Quality control **(QC)** samples were prepared by pooling equal volumes of all experimental samples and were analyzed throughout the run to monitor system stability and instrument performance. Blank samples (53% methanol in water) were processed in parallel to remove background ions. Metabolite types and concentrations were detected using a QExactive™ HF mass spectrometer (Thermo Fisher Scientific, Bremen, Germany) and a Vanquish UHPLC chromatograph (Thermo Fisher Scientific, Bremen, Germany). The raw data were imported into Compound Discoverer 3.1 (Thermo Scientific, Waltham, MA, USA) for spectral processing and database searching to obtain qualitative and quantitative results. Peak areas were normalized using the total area sum method, and metabolites with a coefficient of variation (CV) > 30% in QC samples were excluded. Data transformation and partial least squares discriminant analysis **(PLS-DA)** were performed using metaX software to obtain variable importance in projection **(VIP)** values for each metabolite (Wen et al., 2017). Statistical significance (p-value) of each metabolite between the two groups was calculated based on the t-test, and fold change **(FC)** values were calculated as the ratio of mean levels between groups. Differentially expressed metabolites **(DEMs)** were defined based on the criteria of VIP > 1.0, FC > 1.2 or FC < 0.833, and *P* < 0.05. All identified metabolites were annotated using the KEGG database, and pathways with *P* < 0.05 were considered significantly enriched.

#### Combined transcriptome and metabolome analysis

Pathway enrichment analysis was performed using Metaboanalyst software (www.metaboanalyst.ca) to identify the pathways in which differential metabolites and differential genes were jointly involved in the FH and FL groups; Pearson's correlation coefficient was used to evaluate the correlation between differential metabolites and differential genes in the FH and FL groups.

#### Statistical analysis

Statistical analysis was performed using the software IBM SPSS Statistics version 22 (IBM, Armonk, NY). The content of muscle flavor substances and the cross-sectional area of subcutaneous adipocytes were analyzed by one-way analysis of variance and t-test, respectively. GraphPad Prism 8 software (GraphPad Software Inc., San Diego, CA, USA) was applied for making graph. It was considered to be statistically signifcant when *P*-value <0.05. Novomagic (https://magic.novogene.com) cloud platform tool was used to visualize the correlation between DEGs and DEMs.

## Results

### Muscle flavor substances in the high and low subcutaneous fat groups

As shown in Table 1, there was no significant difference between the FH and FL groups in the amino acid content in the pectoral and leg muscles (*P* > 0.05). Except for Serine, the content of 15 amino acids such as aspartate, threonine, and glycine in the pectoral muscle of FH and FL groups was significantly higher than that of the leg muscle (*P* < 0.05). As shown in Table 2, the content of C20:4n6 in the pectoral muscle of the FH group was significantly lower than that of the FL group (*P* < 0.05), and C16:0, C16:1n7, C18:0, C18:1n9c, C18:2n6c, C20:4n6, C22:6n3, SFA, UFA, PUFA, and MUFA in the leg muscle were significantly higher than that of the FL group (*P* < 0.05). The fatty acid content in the leg muscles showed an overall trend to be higher than that of the pectoral muscles, with C16:0, C16:1n7, C18:0, C18:1n9c, UFA, PUFA, and MUFA being significantly higher in the leg muscles than in the pectoral muscles (*P* < 0.05).

**Table 1:** Comparison of muscle amino acid content (g/100 g) between high and low subcutaneous fat deposition groups of baicheng oil chicken.

Items	Pectoral muscle		Leg muscle	
	FH	FL	FH	FL
Aspartate	2.24±0.08^a^	2.23±0.06^a^	2.03±0.06^b^	2.04±0.06^b^
Threonine	1.06±0.03^a^	1.05±0.05^a^	0.99±0.04^b^	0.99±0.03^b^
Serine	0.90±0.05^ab^	0.91±0.02^a^	0.89±0.03^ab^	0.87±0.03^b^
Glutamate	3.48±0.13	3.51±0.09	3.45±0.10	3.48±0.11
Glycine	1.00±0.03^a^	0.97±0.03^a^	0.89±0.03^b^	0.91±0.04^b^
Alanine	1.35±0.05^a^	1.34±0.03^a^	1.22±0.04^b^	1.21±0.03^b^
Valine	1.18±0.04^a^	1.14±0.04^a^	1.00±0.04^b^	1.01±0.04^b^
Isoleucine	1.10±0.02^a^	1.09±0.03^a^	0.98±0.03^b^	0.98±0.04^b^
Leucine	1.90±0.04^a^	1.89±0.05^a^	1.74±0.05^b^	1.75±0.05^b^
Tyrosine	0.87±0.07^a^	0.85±0.04^ab^	0.81±0.03^b^	0.84±0.04^ab^
Phenylalanine	0.99±0.03^a^	0.98±0.04^a^	0.88±0.03^b^	0.90±0.03^b^
Methionine	0.69±0.03^a^	0.70±0.02^a^	0.65±0.02^b^	0.66±0.02^b^
Proline	0.79±0.05^a^	0.79±0.05^a^	0.74±0.04^b^	0.74±0.03^b^
Arginine	1.53±0.04^a^	1.54±0.04^a^	1.42±0.02^b^	1.42±0.04^b^
Lysine	2.09±0.08^a^	2.08±0.05^a^	1.93±0.05^b^	1.95±0.07^b^
Histidine	1.01±0.11^a^	1.04±0.17^a^	0.75±0.13^b^	0.68±0.05^b^
ΣTotal amount of 16 amino acids	22.18±0.67^a^	22.09±0.44^a^	20.35±0.57^b^	20.40±0.59^b^
ΣFlavor amino acids	9.60±0.30^a^	9.59±0.24^a^	9.00±0.22^b^	9.05±0.25^b^
ΣAromatic amino acids	2.22±0.12^a^	2.19±0.07^a^	2.03±0.06^b^	2.05±0.07^b^
ΣFresh amino acids	14.11±0.38^a^	14.06±0.34^a^	13.13±0.34^b^	13.20±0.34^b^

Note: ΣFlavor amino acids, sum of aspartic acid, glutamic acid, glycine, alanine, and arginine; ΣAromatic amino acids, sum of alanine and tyrosine; ΣFresh amino acids, sum of aspartic acid, serine, glutamic acid, glycine, valine, isoleucine, leucine, proline, and arginine; ΣEssential amino acid, sum of threonine, valine, isoleucine, leucine, phenylalanine, methionine, arginine, and lysine; ΣNon essential amino acids, sum of aspartic acid, serine, glutamic acid, glycine, alanine, tyrosine, proline. Statistical differences (*P* < 0.05) are denoted with different letters (a, b).

**Table 2:** Comparison of muscle fatty acid content (mg/100 g) between high and low subcutaneous fat deposition groups of baicheng oil chicken.

Items	Pectoral muscle		Leg muscle	
	FH	FL	FH	FL
C14:0	3.41±0.07^b^	4.5 ± 0.31^b^	7.37±1.61^a^	5.41±1.60^ab^
C16:0	135.61±32.02^c^	135.36±46.5^c^	289.60±54.10^a^	208.40±62.46^b^
C16:1n7	18.35±5.65^c^	16.96±9.22^c^	56.3 ± 15.63^a^	37.97±15.23^b^
C18:0	53.51±13.10^c^	55.80±12.69^c^	122.37±15.34^a^	87.80±18.47^b^
C18:1n9c	164.30±45.95^c^	161.80±63.61^c^	369.80±68.81^a^	283.00±97.32^b^
C18:2n6c	58.60±15.14^c^	61.55±18.7^c^	167.70±23.28^a^	115.42±30.63^b^
C20:3n6	4.41±0.67	3.57±1.32	4.88±0.75	3.73±0.49
C20:4n6	49.23±10.52^c^	57.09±6.63^b^	77.41±7.47^a^	48.50±5.26^c^
C22:6n3	6.78±2.00^b^	7.17±1.79^b^	9.31±2.16^a^	5.49±1.20^b^
ΣSFA	240.26±38.47^b^	288.60±52.78^b^	419.34±68.94^a^	311.24±80.04^ab^
ΣUFA	341.07±66.03^c^	305.37±54.99^c^	710.32±72.69^a^	434.17±77.07^b^
ΣPUFA	134.87±18.49^c^	133.04±17.60^c^	259.30±28.75^a^	163.50±20.26^b^
ΣMUFA	161.75±27.61^c^	160.41±46.31^c^	426.10±82.81^a^	300.81±26.19^b^

Note: ΣSFA, sum of myristic acid, palmitic acid, and stearic acid; ΣUFA, sum of palmitic acid, oleic acid, linoleic acid, eicosaterionic acid, eicosatetraenoic acid, and eicosadehexaenoic acid; ΣPUFA, sum of polyunsaturated fatty acids includes linoleic acid, eicosaterionic acid, eicosatetraenoic acid, and eicosadehexaenoic acid; ΣMUFA, sum of palmitic oleic acid and oleic acid. Statistical differences (*P* < 0.05) are denoted with different letters (a, b, c).

### Morphological analysis of liver and subcutaneous adipose tissue

**Fig. 1:**
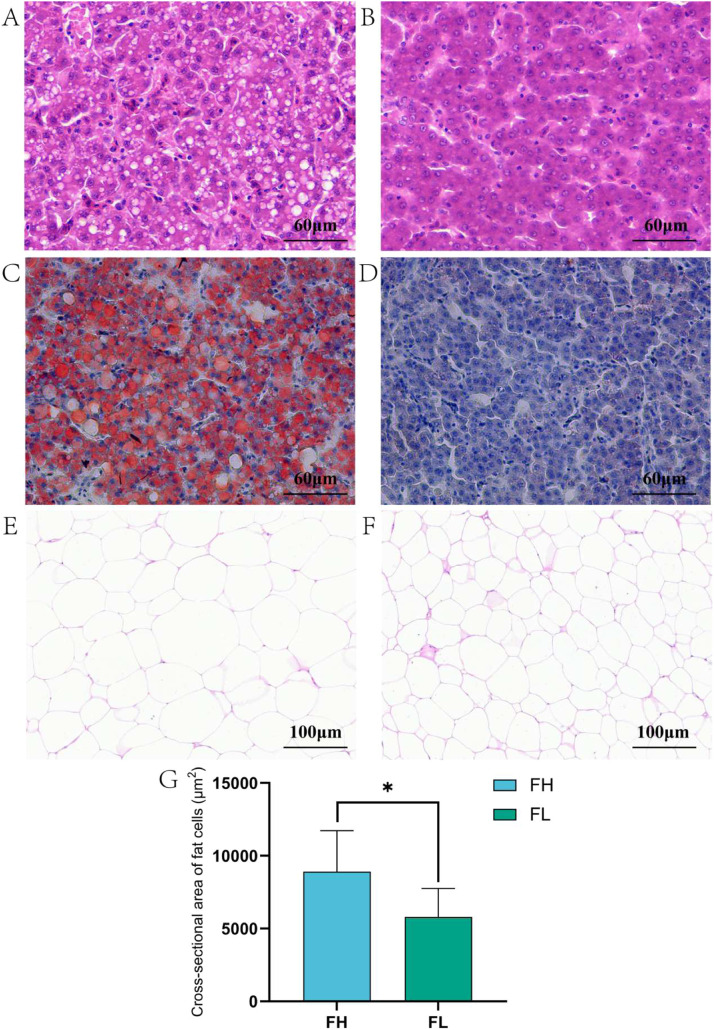
Morphological analysis of liver and subcutaneous adipose tissue in FH and FL groups. (A) Hematoxylin-eosin (HE) stained section of liver in FH group. (B) HE stained section of liver in FL group. (C) Oil red O-stained sections of liver of FH group. (D) Oil red O-stained sections of liver of FL group (E) HE-stained section of subcutaneous fat in FH group. (F) HE-stained section of subcutaneous fat in FL group. (G) Comparison of subcutaneous adipocyte cross-sectional area between FH and FL groups. T-test was used to compare the cross-sectional areas of subcutaneous fat cells between the FH group and the FL group. * *P* ≤ 0.05.

The results of HE stained sections of the liver showed that the cytoplasm of the liver in the FH group presented many lipid vacuoles of different sizes, and some nuclei were extruded to the edge of the cells (Fig. 1A). In the FL group, the hepatocytes were neatly arranged, with a clear structure, and the nuclei of the cells were not obviously extruded, and there were no obvious lipid vacuoles (Fig. 1B). The results of liver oil red O-stained sections showed that the hepatocytes in the FH group showed red or orange colour (Fig. 1C); the hepatocytes in the FL group showed blue colour as a whole, with a small amount of red or orange spots interspersed (Fig. 1D). Observation of subcutaneous adipose tissue sections revealed that the cross-sectional area of adipocytes in the FH group was significantly larger than that in the FL group (Fig. 1E-G).

### Differential gene analysis and functional enrichment

**Fig. 2:**
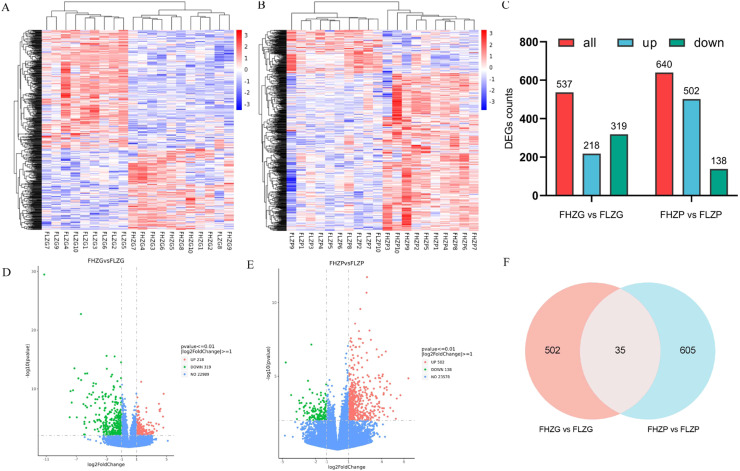
Differential gene analysis of liver and subcutaneous fat in FH and FL groups. (A) Heatmap of differential genes in the FHZG and FLZG groups. (B) Heatmap of differential genes in the FHZP and FLZP groups. (C) Histogram of the number of differential genes in the two comparison groups. Abbreviation: DEGs, differentially expressed genes‌. (D) Volcano map of differential genes in the FHZG and FLZG groups. (E) Volcano map of differential genes in the FHZP and FLZP groups. (F) Venn diagram of shared differential genes in the two comparison groups.

In this study, RNA-seq data of 40 samples of liver and subcutaneous fat from FH and FL groups were analysed, and it was found that the clean date totalled 245.39 Gb, with Q30 above 88.23% and GC content between 48.49% and 52.08% (Table S2-1). In addition, the Reads of 40 samples were compared to the reference genome, and the comparison rate was above 89.98% (Table S2-2). In the sample correlation analysis, it was found that the FH and FL groups of Baicheng oil chicken were both highly correlated within the groups with similar expression patterns, and the gene expression differences between the groups were large, indicating that the sample selection was reasonable (Figs. 2A-B). As shown in Fig. 2C, 537 and 640 DEGs with significant expression were identified in liver and subcutaneous adipose tissue respectively. In liver tissue, 218 genes were significantly up-regulated and 319 genes were significantly down regulated (Fig. 2D, and Table S3-1). In subcutaneous adipose tissue, there were 502 significantly up-regulated and 138 significantly down regulated genes (Fig. 2E, and Table S3-2). A total of 35 genes are common between liver and subcutaneous adipose tissue (Fig. 2F, and Table S4).

**Fig. 3:**
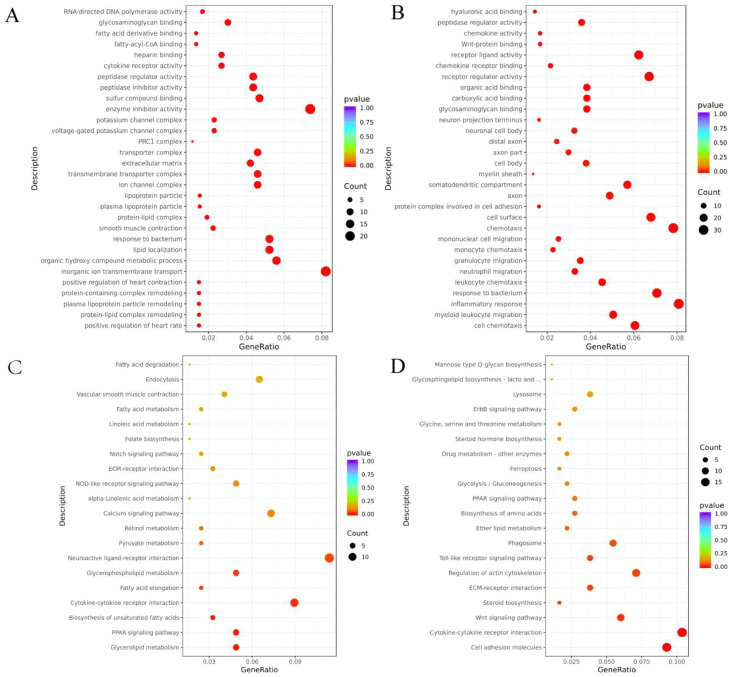
Functional annotation analysis of DEGs among different groups. (A) Gene Ontology (GO) enrichment analysis of DEGs between FHZG and FLZG. (B) GO enrichment analysis between FHZP and FLZP. (C) Kyoto Encyclopedia of Genes and Genomes (KEGG) enrichment analysis of DEGs between FHZG and FLZG. (D) KEGG enrichment analysis between FHZP and FLZP.

**Fig. 4:**
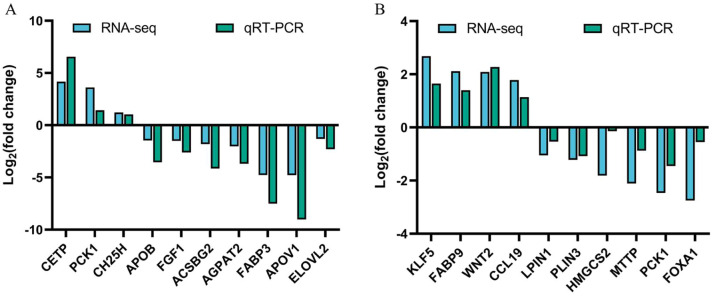
Comparison of RNA-seq and qRT-PCR gene expression in liver and subcutaneous adipose tissue between FH and FL groups of Baicheng oil chickens. (A) Comparison of gene expression by RNA-seq and qRT-PCR between FHZG and FLZG. (B) Comparison of gene expression by RNA-seq and qRT-PCR between FHZP and FLZP.

Subsequently, we performed GO and KEGG enrichment analyses for all differential genes (Fig. 3) and found that differentially expressed genes were significantly enriched in 106 GO terms (*P* < 0.01) in liver tissues of Baicheng oil chickens, including the entries of protein-lipid complex remodeling, plasma lipoprotein particle remodeling, and lipid localisation entries (Fig. 3A and Table S5-1). In the subcutaneous adipose tissue of Baicheng oil chickens, differentially expressed genes were significantly enriched in 276 GO terms (*P* < 0.01), including cell chemotaxis, protein-lipid complex remodeling, cellular response to lipopolysaccharide and other entries (Fig. 3B and Table S5-2). In addition, KEGG enrichment analysis showed that DEGs were significantly enriched in glyceride metabolism, PPAR signaling pathway, and unsaturated fatty acid biosynthesis pathway in liver tissues of Baicheng oil chickens (*P* < 0.01) (Fig. 3C and Table S6-1). In the subcutaneous adipose tissue of Baytown oil chicken hens, DEGs were significantly enriched in cell adhesion molecules cytokine-cytokine receptor interaction pathway (*P* < 0.01) (Fig. 3D and Table S6-2). Ten differentially expressed genes (DEGs) were randomly selected from the transcriptome sequencing results of liver and subcutaneous adipose tissue each for RT-qPCR. The results showed that the 10 DEGs selected from each of the liver and subcutaneous adipose were consistent with the trend of the qRT-PCR results (Fig. 4), indicating that the results of RNA-seq were accurate and reliable.

### Differential metabolite analysis and functional enrichment

**Fig. 5:**
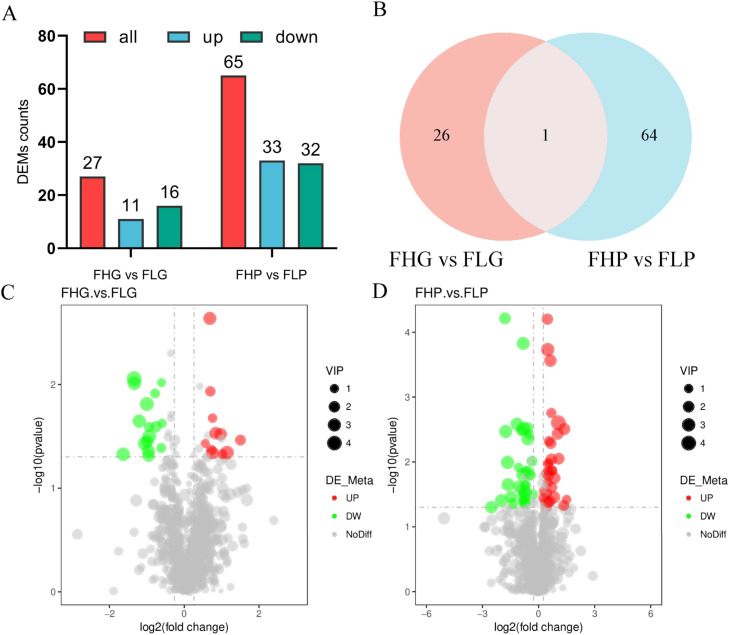
Differential metabolite analysis of liver and subcutaneous fat in FH and FL groups. (A) Histogram of the number of differential metabolites for the two comparison groups. Abbreviation: DEMs, differentially expressed metabolites‌. (B) Venn diagram of shared differential genes in the two comparison groups. (C) Volcano plot of differential metabolites between FHG and FLG groups. (D) Volcano plot of differential metabolites between FHP and FLP groups.

In this study, through metabolomics analysis and quantitative analysis of 40 samples of liver and subcutaneous fat from the FH and FL groups of Baicheng oil chickens, a total of 678 metabolites in positive ion mode and 375 metabolites in negative ion mode were identified, including lipids and lipid-like molecules, organic acids and derivatives The samples were analysed by metabolomics, and 678 positive-mode metabolites and 375 negative-mode metabolites were identified, including lipids and lipid-like molecules, organic acids and derivatives, nucleosides, nucleotides, etc. (Fig.s S1A−*B*). PLS-DA analysis showed that metabolites were significantly separated between sample groups, indicating that metabolites differed between liver and subcutaneous adipose tissue of Baicheng oil chickens in FH and FL groups (Fig.s S1C-D). As shown in Fig. 5A, there were 27 DEMs between the FHG and FLG groups, of which 11 metabolites were up regulated and 16 metabolites were down regulated, and 65 DEMs between the FHP and FLP groups, of which 33 metabolites were up regulated and 32 metabolites were down regulated (VIP > 1.0, FC > 1.2 or FC < 0.833 and *P*-value < 0.05). Further analysis revealed that the differential metabolite adenosine was shared between the liver and subcutaneous adipose tissue (Fig. 5B). Compared to the FHG group, pinocembrin, chrysin, and galangin contents were significantly higher in the FLG group, while uridine diphosphate galactose, beta-Nicotinamide adenine dinucleotide phosphate,and N-Isovalerylglycine contents were clearly lower (Fig. 5C and Table S7-1). Compared to the FHP group, N-Acetyl-1-aspartylglutamic acid, ACar 20:2, and ACar 16:1 contents were significantly higher in the FLP group, while troxerutin, 4,5-Dicaffeoylquinic acid, and theobromine were clearly lower (Fig. 5D and Table S7-2).

**Fig. 6:**
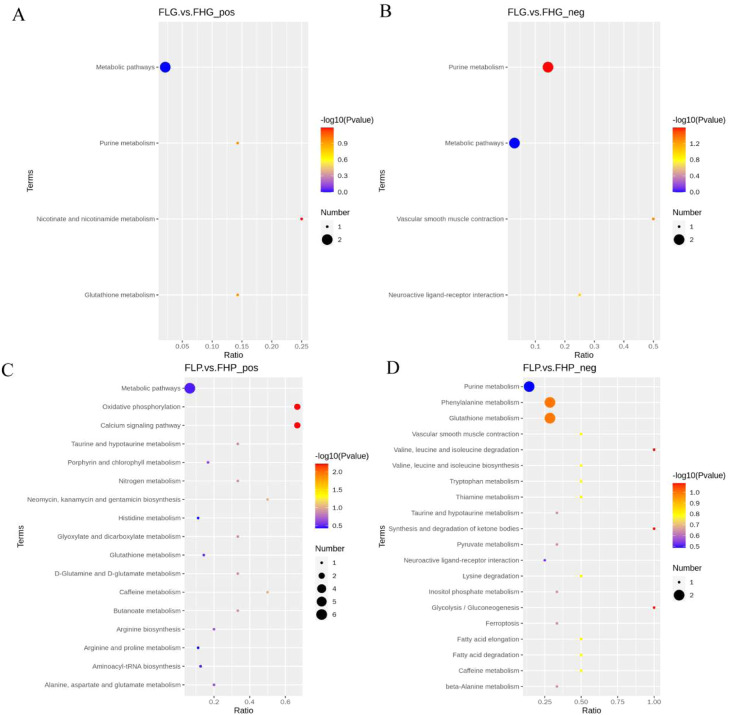
Functional annotation analysis of DEMs among different groups. (A) KEGG enrichment analysis of DEMs between FHG and FLG in positive ion mode. (B) KEGG enrichment analysis of DEMs between FHG and FLG in negative ion mode. (C) KEGG enrichment analysis of DEMs between FHP and FLP in positive ion mode. (D) KEGG enrichment analysis of DEMs between FHP and FLP in negative ion mode.

Subsequently, we analysed KEGG enrichment of differential metabolites between liver and subcutaneous adipose tissues of Baicheng oil chickens from FH and FL groups (Fig. 6). In the liver tissues of Baicheng oil chickens hens, DEMs were significantly enriched in the purine metabolism, and vascular smooth muscle contraction pathways (*P* < 0.05) (Fig.s 6A-B and Table S8-1). In the subcutaneous adipose tissue of Baicheng oil chickens, DEMs were significantly enriched in the oxidative phosphorylation, and calcium signaling pathway (*P* < 0.05) (Fig.s 6C-D and Table S8-2).

### Combined Analysis of Differential Genes and Differential Metabolites

**Fig. 7:**
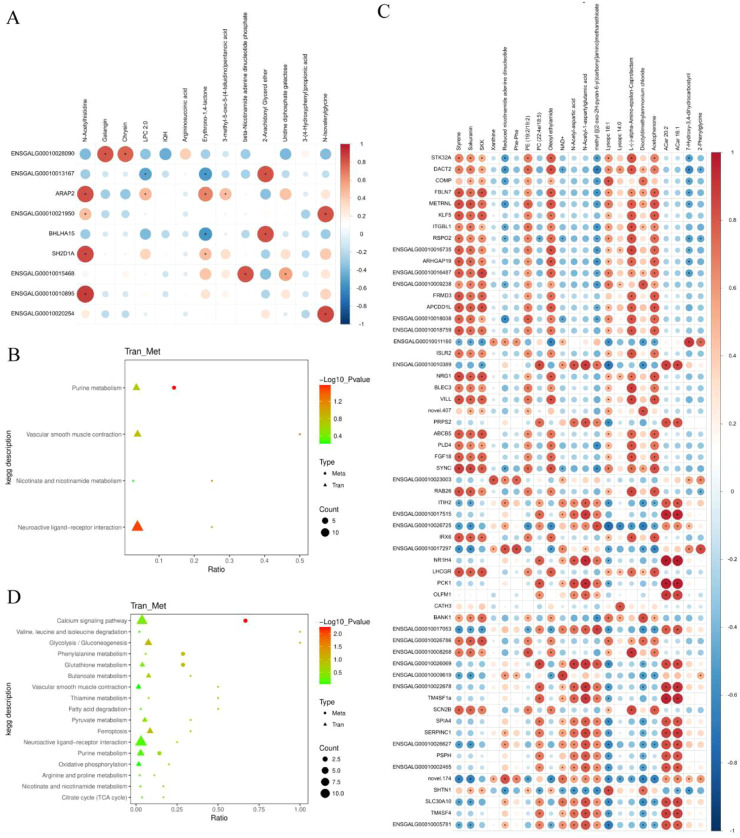
Analysis of DEGs and DEMs co-enrichment pathways and their correlations. (A) Correlation analysis of DEGs and DEMs between FHG and FLG. The correlation analysis heatmap illustrates the correlations between differentially expressed genes and various differentially expressed metabolites. Using Pearson's correlation method, we calculated the correlation coefficients and p-values between each differentially expressed gene and the various differentially expressed metabolites. The x-axis represents differentially expressed metabolites, the left y-axis represents differentially expressed genes, and the right y-axis shows the correlation coefficients; blue indicates a negative correlation, while red indicates a positive correlation. *P ≤ 0.05. (B) DEGs and DEMs co-enrichment pathway between FHG and FLG. (C) Correlation analysis of DEGs and DEMs between FHP and FLP. (D) DEGs and DEMs co-enrichment pathway between FHP and FLP.

In this study, we performed correlation analyses of differential genes and differential metabolites in liver and subcutaneous fat in FH and FL groups of Baicheng oil chickens, and identified three positively correlated gene-metabolite pairs and six negatively correlated gene-metabolite pairs in the liver group at *P* < 0.05 and correlation coefficients >0.8 (Fig. 7A), and together they are involved in the pathways of nicotinate and nicotinamide metabolism, purine metabolism, and neuroactive ligand-receptor interaction (Fig. 7B). One hundred and nineteen positively correlated gene-metabolite pairs and two negatively correlated gene-metabolite pairs were identified in the subcutaneous adipose group (Fig. 7C), and were collectively involved in the pathways of nicotinate and nicotinamide metabolism, purine metabolism, purine metabolism, and neuroactive ligand-receptor interaction (Fig. 7D).

## Discussion

The flavor of chicken meat consists mainly of the taste and aroma of the meat, both of which originate from flavor precursors in raw meat. The odor perceived by the sense of smell is mainly composed of volatile chemicals, which are mostly derived from nutrients such as amino acids and fatty acids (Gonda et al., 2018). Amino acids serve as key indicators for assessing meat quality, with their types and content directly influencing the nutritional value and flavour of poultry meat (Luo et al., 2022). In this study, there was no significant difference in the amino acid content in breast and leg muscles between the groups of Baicheng oil chickens with high and low subcutaneous fat deposition, indicating that subcutaneous fat deposition has less effect on muscle amino acid synthesis in Baicheng oil chickens. It was found that muscle flavor was closely related to lipids and volatile flavor compounds (Hunt et al., 2016). In the comparison of the amino acid content of the breast muscle and leg muscle, it was found that the content of 15 amino acids, such as aspartate, threonine, and glycine in the breast muscle was significantly higher than that of the leg muscle, indicating that the breast muscle of the Baicheng oil chicken was superior to the leg muscle in terms of amino acid composition, which provided a material basis for the muscle flavor of the breast muscle of the Baicheng oil chicken.

Fatty acid composition is an important indicator of food stability and nutritional value (Nid et al., 2024). Fatty acids can be classified into saturated and unsaturated fatty acids, and meat with high levels of saturated and monounsaturated fatty acids has a good texture and strong flavour (Damaziak et al., 2019). The different fatty acid composition of muscle is likely to affect lipid stability and flavour (Chen et al., 2022). In this study, the C20:4n6 content in the pectoral muscle of the FH group was significantly lower than that of the FL group, and the C16:0, C16:1n7, C18:0, C18:1n9c, C18:2n6c, C20:4n6, C22:6n3, SFA, UFA, PUFA, and MUFA in the leg muscles were significantly higher than that of the FL group. Moreover, the fatty acid content in the leg muscles showed an overall trend of being higher than that in the pectoral muscles, with C16:0, C16:1n7, C18:0, C18:1n9c, UFA, PUFA, and MUFA being significantly higher in the leg muscles than in the pectoral muscles. It indicated that subcutaneous fat deposition affected fatty acid synthesis and catabolism to a certain extent and that high subcutaneous fat deposition promoted fatty acid synthesis in the leg muscle of the Baicheng oil chicken.

The liver is an important organ for metabolism and lipid synthesis, catabolism, and transport processes in the poultry organism (Aierken et al., 2022). Adipose tissue cannot expand through adipocyte proliferation, which can lead to adipocyte hypertrophy (Ting et al., 2023). Chen et al. (2023) found that the liver lipid vacuole area of Beijing ducks fed high-fat diet was higher than that of Beijing ducks fed low-fat diet, indicating that the liver lipid deposition of Beijing ducks fed high-fat diet was higher than that of Beijing ducks fed low-fat diet. Zhang et al. (2022) In comparing lipid metabolism levels and intestinal microflora in chickens of different body weights at the same day of age, it was found that chickens with higher body weights had more fat in their livers, and their fat deposits were greater. Liu et al. (2023) found that the cross-sectional area of chicken abdominal adipocytes gradually increased with increasing lipid accumulation. In this study, it was found that the liver cytoplasm of the FH group showed a large number of lipid vacuoles, and the hepatocytes showed a large number of red or orange colors, indicating that their hepatocytes contained a large number of lipid droplets; the liver of the FL group had no obvious lipid vacuoles, and the hepatocytes as a whole showed a blue color, with a small number of interspersed red or orange patches, which indicated that their hepatocytes contained a lower amount of lipids. In addition, the cross-sectional area of fat cells in the FH group was significantly larger than that in the FL group. The combined results of liver and subcutaneous adipose tissue morphology indicated that the FH group promoted subcutaneous fat deposition by increasing the number of lipid droplets and increasing the cross-sectional area of fat cells.

The liver of poultry plays an important role in fat metabolism and is the key metabolic organ of fat synthesis and catabolism (Cao et al., 2022). Transcriptome sequencing can be used to analyse gene expression differences between groups under specific physiological conditions, thereby further investigating the regulatory mechanisms and functional roles of genes, and has been widely used in the screening of genes related to lipid metabolism in animals (Luo et al., 2022; Zhao et al., 2022). Through transcriptome analysis of Baicheng oil chicken liver, a total of 537 highly expressed DEGs were identified, which were significantly enriched in glyceride metabolism pathway, PPAR signaling pathway, unsaturated fatty acid biosynthesis and other pathways related to lipid metabolism. Moreover, apolipoprotein B **(APOB)**, apovitellenin 1 **(APOV1)**, membrane bound glycerophospholipid O-acyltransferase 2 **(MBOAT2)**, lipin 2 **(LPIN2)**, 1-acylglycerol-3-phosphate O-acyltransferase 2 **(AGPAT2)**, fatty acid binding protein 3 **(FABP3)**, acyl-CoA synthetase bubblegum family member 2 **(ACSBG2)**, PCK1 and acetyl-CoA acyltransferase 1 **(ACAA1)** related to lipid metabolism were screened. APOB belongs to the apolipoprotein family and can play an important role in lipid metabolism by affecting the metabolism of TG and cholesterol (Su and Peng, 2020). APOV1 is synthesised in the liver, and it was found to be highly expressed in the liver of laying hens, where it promotes the synthesis of TG, cholesterol, and very low-density lipoproteins (Liu, et al., 2019). In this study, the differential genes APOB and APOV1 were enriched in glyceride metabolism pathway and unsaturated fatty acid biosynthesis pathway, and were highly expressed in liver tissues of low subcutaneous fat deposition group. These results suggest that APOB and APOV1 play an important role in liver lipid metabolism of Baicheng oil Chickens by affecting the biosynthesis of triglycerides and unsaturated fatty acids. MBOAT2, LPIN2, and AGPAT2 can affect triglyceride biosynthesis by participating in glycerophospholipid metabolism (Mak et al., 2021; Yang et al., 2019). In this study, the differential genes MBOAT2, LPIN2, and AGPAT2 were significantly enriched in the glycerol ester metabolism pathway, and it is hypothesized that the above genes regulate lipid metabolism in the livers of Baicheng oil chickens by participating in the glycerol ester metabolism pathway. In this study, the expression levels of FABP3, ACSBG2, PCK1, and ACAA1 were significantly different between groups and were enriched in the PPAR signaling pathway. Specifically, FABP3 and ACSBG2, which are involved in fatty acid transport and metabolism (Bi et al., 2020; Guo et al., 2018), showed higher expression in the low subcutaneous fat deposition group. In contrast, PCK1, which promotes triglyceride production and fat deposition by encoding regulatory enzymes for glycerol production (Vieira et al., 2017), and ACAA1, a key enzyme in fatty acid β-oxidation (Huang et al., 2015), were upregulated in the high subcutaneous fat deposition group. These expression patterns suggest that the PPAR signaling pathway may differentially regulate genes related to lipid uptake and oxidation in the liver of Baicheng oil chickens, corresponding to distinct phenotypes of fat deposition.

A variety of transcription factors and genes regulate fat deposition. Guo et al. (2021) used RNA-seq to identify differentially expressed genes in subcutaneous fat at different developmental stages of ducks. They found that compared to 12 weeks of age, subcutaneous adipose tissue of ducks at 35 weeks of age up-regulated genes related to cholesterol biosynthesis and fatty acid biosynthesis, such as hydroxysteroid 17-beta dehydrogenase 7 **(HSD17B7)** and methylsterol monooxygenase 1 **(MSMO1)**, and down-regulated fatty acids β-oxidation-related genes such as acyl-CoA oxidase 1 **(ACOX1)** and acyl-CoA synthetase long chain family member 1 **(ACSL1)**. In this study, 640 significant DEGs were identified by transcriptome analysis of the subcutaneous adipose tissue of Baicheng oil chicken. The above DEGs were significantly enriched in pathways related to lipid metabolisms, such as cell adhesion molecules, cytokine-cytokine receptor interactions, and other pathways, and candidate genes related to lipid metabolisms, such as Wnt family member 2 **(WNT2)**, C—C motif chemokine ligand 19 **(CCL19)**, cathepsin K **(CTSK)**, lipase A, lysosomal acid type **(LIPA)**, periostin **(POSTN)**, and phospholipid transfer protein **(PLTP)**, were screened out. The Wnt signaling pathway is associated with adipocyte differentiation and proliferation, and the expression of genes such as WNT2 and transcription factor 7 like 2 **(TCF7L2)** in this pathway can promote adipocyte proliferation (Chen and Wang, 2018). In the present study, WNT2 expression was significantly upregulated in the FH group, and WNT2 was significantly enriched in the Wnt signaling pathway. These results are consistent with the established role of Wnt signaling in promoting adipocyte proliferation, suggesting a possible association between WNT2 upregulation and subcutaneous fat deposition in Baicheng oil chickens. Similarly, the differential genes CCL19, CTSK, LIPA, and POSTN were found to have a role in promoting fat deposition. The expression level of CCL19 was found to be significantly higher in adipose tissue of obese individuals than in lean individuals (Kochumon et al., 2021). The expression of CTSK in adipose tissue was positively correlated with the level of adipose deposition (Fontanesi et al., 2010). LIPA encodes lysosomal lipase, which hydrolyses cholesterol and TG and produces free fatty acids (Puchalska and Crawford, 2017). POSTN is produced in the adipose mesenchymal stromal cells and positively regulates the production of TG (Evans et al., 2019). In this study, CCL19 was significantly enriched in the cytokine-cytokine receptor interaction pathway, and CTSK and LIPA were enriched in the lysosomal pathway, so it was hypothesised that the differential genes CCL19, CTSK, LIPA, and POSTN, might play a positive regulatory role in the process of subcutaneous fat deposition in the Baicheng oil chickens through the lysosomal pathway, etc. The expression of POSTN was upregulated in the FH group, suggesting that POSTN may promote subcutaneous fat deposition in Baicheng oil chickens by positively regulating TG production. In addition, differential genes PLTP and HMGCS2 have also screened in the subcutaneous fat of Baicheng oil chickens co-enriched in the PPAR signaling pathway. PLTP mediates the transfer of phospholipids and free cholesterol (Zhang et al., 2018). The protein encoded by HMGCS2 catalyzes the ketogenic reaction, which provides lipid-sourced energy to various organs in the event of a carbohydrate deficiency in the organism (Pasta et al., 2021). Therefore, it is hypothesized that PLTP and HMGCS2 play a regulatory role in subcutaneous fat metabolism in the Baicheng oil chicken through the PPAR signaling pathway.

Metabolomics is the sum of all low molecular weight metabolites produced in a given organism or cell at a given biological stage and is used to describe the interactions of these metabolites within the organism and the organism's response to the external environment (Zhang et al., 2022). Metabolomic analyses help to provide insight into the differences between lipid deposition and muscle metabolites in animals and facilitate biomarker discovery (Cui et al., 2024; Xu et al., 2024). In this study, metabolomic analysis and quantitative analysis of liver and subcutaneous fat samples of Baicheng oil chickens in FH and FL groups revealed that metabolites were significantly separated between the sample groups, suggesting that metabolites have a certain correlation with fat deposition. The liver is a central organ for lipid metabolism (Zhao et al., 2023). In this study, DEMs of amino acids, fatty acids, and purine nucleosides were identified in the liver tissues of Baicheng oil chickens of the FH and FL groups and were involved in pathways such as purine metabolism and vascular smooth muscle contraction. Studies have shown that abnormalities in histidine metabolism exacerbate oxidative stress, which can lead to lipid accumulation and lipid peroxidation (Zhu et al., 2022). In addition, histidine can improve lipid metabolism by regulating the expression of genes related to cholesterol metabolism and promoting cholesterol clearance and HDL remodeling (Zheng et al., 2023). In the present study, the content of N-acetylhistidine was higher in the liver tissues of the FH group. This metabolite is an acetylated derivative of histidine, and its elevated levels were observed in conjunction with increased fat deposition in the FH group, suggesting a possible association between altered histidine metabolism and lipid accumulation in Baicheng oil chickens. Chrysin is a natural flavonoid that has been shown to influence lipid synthesis and catabolic processes by regulating the expression of genes related to lipid metabolism (Garg and Chaturvedi, 2022). In the present study, chrysin was more abundant in the FL group, which exhibited lower fat deposition. Transcriptomic analysis revealed that multiple lipid metabolism-related genes were differentially expressed between groups. However, the interaction and regulatory mechanisms between chrysin and these differentially expressed lipid metabolism-related genes require further functional validation. In the present study, 65 DEMs of amino acids, fatty acids, and steroids were found in the subcutaneous adipose tissue of Baicheng oil chickens of FH and FL groups and were significantly enriched in the oxidative phosphorylation and calcium signaling pathway. Studies have shown that glycerophospholipids have a key role in lipid deposition and metabolism. For example, in different tissues of chickens, the ELOVL gene family regulates lipid deposition by affecting the composition of glycerophospholipids, particularly by increasing the proportion of long-chain unsaturated glycerophospholipid molecules in muscle, thereby promoting intramuscular fat deposition (Wang et al., 2022). In the present study, glycerophospholipid substances such as lysopc 14:0, lysopc 18:1, and lysopc 18:2 were screened in the subcutaneous adipose tissue of Baicheng oil chickens in the FH and FL groups, and all of them were higher in the high subcutaneous fat deposition group. It is hypothesised that glycerophospholipids such as lysopc 14:0, lysopc 18:1, and lysopc 18:2 promote subcutaneous fat deposition by increasing the proportion of long-chain unsaturated glycerophospholipid molecules. In addition, the present study found that acylcarnitines such as ACar 16:1 and ACar 20:2 were higher in the low subcutaneous fat deposition group. Carnitine and acylcarnitines are usually involved in lipid transport and metabolic processes (Bjorkblom et al., 2022), and an increase in acylcarnitines facilitates lipid oxidation energy production and thus decreases lipid synthesis (Marian et al., 2023). It is thus hypothesized that higher levels of acylcarnitines such as ACar 16:1 and ACar 20:2 promote lipid oxidation and decrease fat deposition. In the present study, the differential metabolite adenosine was detected in both liver and subcutaneous fat of FH and FL groups.Interestingly adenosine was significantly higher in liver tissue of FH group than FL group, however adenosine was significantly lower in subcutaneous adipose tissue of FH group than FL group. Adenosine is an endogenous nucleoside molecule widely involved in energy metabolism, signaling, and inflammation regulation, and its role in lipid deposition is complex. Gnad et al. first found that adenosine activates brown adipose tissue via the A2A receptor, which promotes thermogenesis and lipolysis (Gnad et al., 2014). It has also been suggested that an increase in adenosine may lead to changes in the intracellular level of ATP, which affects fatty acid synthesis and catabolic processes, and this alteration in energy metabolism may lead to the accumulation of lipids in the cell (Ran et al., 2021). The Adenosine content in the subcutaneous adipose tissue of the FL group was significantly higher than that of the FH group in the present study, which may be attributed to the activation of adipose tissue by adenosine through the A2A receptor, which promotes the process of lipolysis.

Based on combined transcriptomic and metabolomic analyses, several differential gene-metabolite pairs were identified in the liver and subcutaneous adipose tissue of the FH and FL groups. For example, ARAP2 and N-acetylhistidine showed a strong positive correlation. N-acetylhistidine, an acetylated derivative of histidine, has been reported to accelerate lipid deposition, potentially through regulating genes involved in cholesterol metabolism. Additionally, knockdown of ARAP2 has been shown to reduce lipid droplet formation and triglyceride synthesis, with further analyses indicating that ARAP2 influences sphingolipid metabolism via glucosylceramide synthase, thereby promoting lipid droplet formation (Chaudhari et al., 2016). In the present study, ARAP2 and N-acetylhistidine were highly positively correlated and both showed higher levels in the liver tissues of the FH group. These observations suggest a potential association between this gene-metabolite pair and lipid deposition in Baicheng oil chickens, warranting further investigation. In addition, PCK1 and the acylcarnitines ACar 16:1 and ACar 20:2 were identified in the subcutaneous adipose tissue of the FH and FL groups, which showed a significant positive correlation. PCK1 can affect triglyceride production by encoding a regulatory enzyme that promotes glycerol production.30 An increase in acylcarnitines facilitates lipid oxidation, and energy production, and thus decreases lipid synthesis (Marian et al., 2023). In this study, PCK1, ACar 16:1, and ACar 20:2 showed significant positive correlation, and both were highly expressed in the subcutaneous adipose tissue of the FL group, and it was hypothesised that PCK1, ACar 16:1, and ACar 20:2 play an important role in lipid metabolism of Baicheng oil chickens through the synergistic regulation of lipid oxidation and metabolism, such as triglycerides. These differential gene-metabolite pairs identified in this study may play important roles in fat deposition in Baicheng oil chickens, providing valuable references for further studies on the molecular mechanisms of fat regulation and meat quality traits in Baicheng oil chickens. In addition, organisms of different germplasm differ in physiological characteristics and developmental processes, leading to differences in metabolism and transcript levels, and subsequent studies will focus on exploring and validating the above candidate genes and metabolites from different growth and developmental time points.

## Conclusions

In this study, Baicheng oil chickens with divergent subcutaneous fat deposition were investigated through integrated transcriptomic and metabolomic analyses of liver and adipose tissues. Key differentially expressed genes involved in lipid metabolism, including APOB, MBOAT2, and PCK1, were identified, along with differentially abundant metabolites such as N-acetylhistidine, chrysin, and lysoPC 14:0. Furthermore, significant gene-metabolite correlations, including ARAP2–N-acetylhistidine, PCK1–ACar 16:1, and PCK1–ACar 20:2, were revealed, which may participate in pathways such as nicotinate and nicotinamide metabolism and purine metabolism to influence lipid metabolic processes. Collectively, these findings provide a comprehensive resource for understanding the regulatory networks underlying fat deposition in local chicken breeds. The identified genes, metabolites, and their interactions serve as potential biomarkers for breeding selection and lay a foundation for further functional studies aimed at improving meat quality traits in Baicheng oil chickens.

## Data availability statement

RNA-Seq data is available at NCBI SRA: PRJNA1436178, and metabolomics data is available at Metabolomics Workbench: ST004702.

## CRediT authorship contribution statement

**Xiaoyu Zhao:** Writing – review & editing, Writing – original draft, Funding acquisition, Formal analysis, Conceptualization. **Yang Yao:** Investigation, Data curation. **Haiying Li:** Project administration, Funding acquisition. **Herong Liao:** Methodology. **Wei Dong:** Resources. **Yingping Wu:** Visualization.

## Disclosures

All authors declare that they have no competing interests.

## Appendix Supplementary materials

Image, application 1

Image, application 2

Image, application 3

Image, application 4

Image, application 5

Image, application 6

Image, application 7

Image, application 8

Image, application 9

## Footnotes

Supplementary material associated with this article can be found, in the online version, at doi:10.1016/j.psj.2026.106894.
