# Supplementary material for: Integrated transcriptomic and metabolomic analyses reveal the regulatory mechanism of subcutaneous fat deposition in Baicheng Oil chickens
Source: Poult Sci. 2026 Apr 2;105(7):106894. doi: 10.1016/j.psj.2026.106894 (PMC13090636; doi:10.1016/j.psj.2026.106894)

Supporting Information

**Integrated Transcriptomic and Metabolomic Analyses Reveal the Regulatory Mechanism of Subcutaneous Fat Deposition in Baicheng Oil Chickens**

Xiaoyu Zhao^a,1^, Yang Yao^a^, Haiying Li^a,*^, Herong Liao^b^, Wei Dong^b^ and Yingping Wu^a^

^a^ College of Animal Science, Xinjiang Agricultural University, Urumqi, 830052, China

^b^ Xinjiang Noach Baicheng Oil Chicken Development Co.,Baicheng, 842300, China

Figure S1. Identification of hepatic and subcutaneous adipose tissue metabolites between the FH and FL groups of Baicheng oil chickens. (A) Pie chart of classification of all metabolites in positive ion mode. (B) Pie chart of the classification of all metabolites in the negative ion mode. (C) Scatterplot of PLS-DA model scores for FHG and FLG groups. (D) Scatterplot of PLS-DA model scores for FHP and FLP groups.


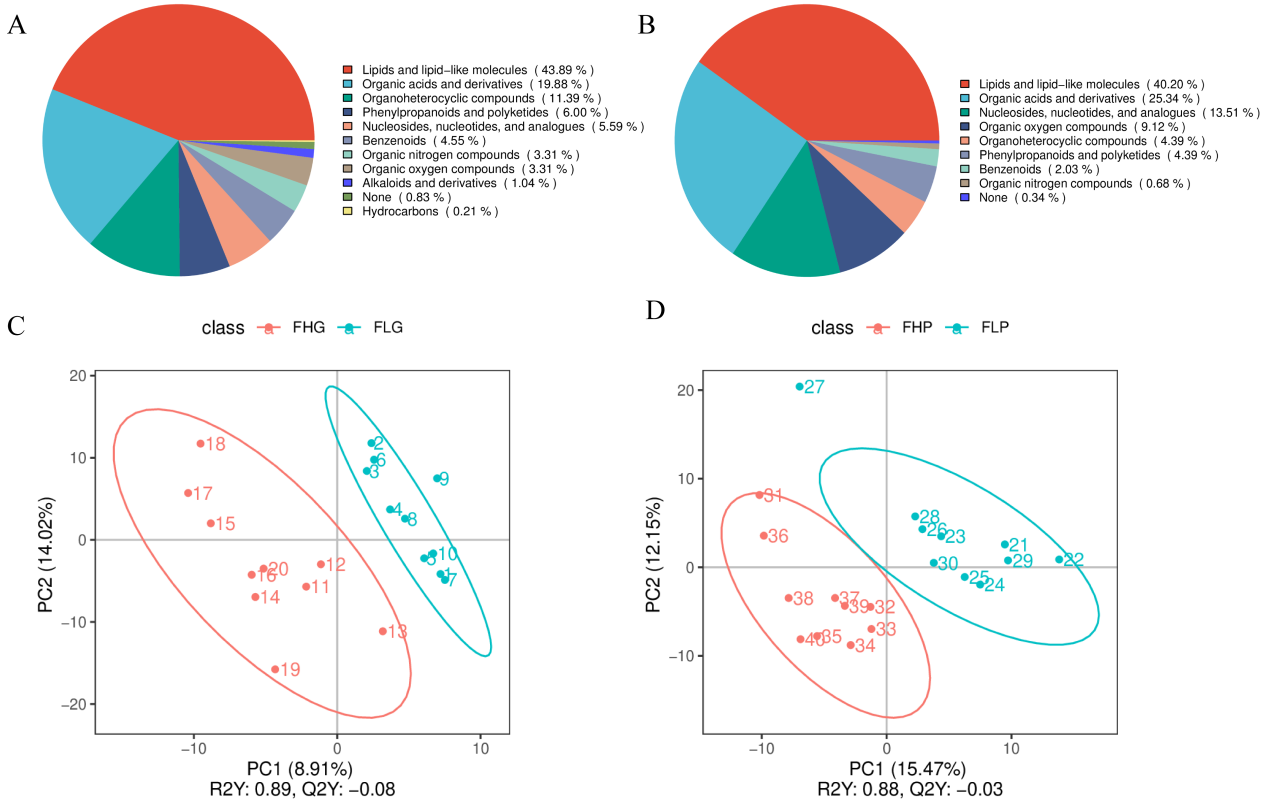

Supplement: Supplementary file 1 [file mmc1.docx]
